# Adipose-Derived Stromal/Stem Cells

**DOI:** 10.3390/cells9091997

**Published:** 2020-08-30

**Authors:** Patrick C. Baer

**Affiliations:** Division of Nephrology, Department of Internal Medicine III, University Hospital, Goethe-University, 60596 Frankfurt am Main, Germany; patrick.baer@kgu.de or p.baer@em.uni-frankfurt.de; Tel.: +49-69-6301-5554; Fax: +49-69-6301-4749

Adipose tissue is a rich, ubiquitous, and easily accessible source for multipotent mesenchymal stromal/stem cells (MSCs), so-called adipose-derived stromal/stem cells (ASCs). Primary isolated ASCs are a heterogeneous preparation consisting of several subpopulations of stromal/stem and precursor cells. Donor-specific differences in ASC isolations and the lack of culture standardization hinder the comparison of results from different studies. Nevertheless, ASCs are already used in different in vivo models and clinical trials to investigate their ability to improve tissue and organ regeneration. Many questions concerning their counterparts and biology in situ, their differentiation potential in vitro and in vivo, and also the mechanisms of regeneration (paracrine effects including regeneration promoting factors and extracellular vesicles, differentiation, immunomodulation) are not completely understood or remain unsolved. For this reason, this special edition aims to expand current knowledge about the extremely diverse potential of ASCs.

This Special Issue covers research articles investigating various adipose tissues as a source for ASC isolation [1,2,3], specific cultures methods to enhance proliferation or viability [4,5,6,7], and the differentiation capacity [8,9,10,11,12]. Furthermore, other studies highlight aspects of various diseases [13,14], the immunosuppressive potential of ASCs and their derivates [15] or the in vivo tracking of transplanted ASCs [16].

Ritter and co-workers analyzed the functional similarities and differences of ASCs isolated from different adipose depots [3]. The authors described that ASCs isolated from subcutaneous and visceral fat share multiple cellular features, but significantly differ in their functions. The functional diversity of ASCs depends on their origin, cellular context, and surrounding microenvironment within adipose tissues. Stojanović and co-workers characterized the molecular signature and the differentiation capacity of ASCs isolated from lipoma [2]. A study by our group from the nephrological research laboratory summarized the isolation and culture of ASCs from perirenal adipose tissue, characterized the cultured cells, and demonstrated their immunomodulatory potential and their high permissiveness for human cytomegalovirus [1].

Platelet lysate has been shown to be an effective replacement for serum in the culture, expansion, and differentiation of ASCs [4,7]. Metformin has been shown as a preconditioning agent that stimulates proliferative activity and viability of ASCs [6]. The addition of metformin improved metabolism and viability, correlated with higher mitochondrial membrane potential, and reduced apoptosis. As a possible alternative to standard cell culture, Ryu and co-workers reviewed spheroid culture systems that could provide a physicochemical environment similar to that in vivo by facilitating cell-cell and cell-matrix interaction, thereby overcoming the limitations of traditional monolayer cell culture [5].

The findings of Ng and co-workers suggest that the epigenetic state of MSCs is associated with the biased differentiation plasticity towards its tissue of origin, proposing a mechanism related to the retention of epigenetic memory [11]. This result could improve the selection of optimal tissue sources for MSCs for therapeutic applications. Others studied various effects of differentiation events induced by differentiation-inducing agents. Using valproic acid, the induced neural differentiation of ASCs was demonstrated by the upregulation of characteristic neuro-specific factors [8]. Zöller and co-workers showed that collagen I was able to modulate lipogenesis and adiponectin expression, and hypothesized that this could contribute to age-related metabolic disorders [10]. Klemenz and co-workers determined volatile organic compounds during adipogenic differentiation of ASCs in order to avoid cell destruction during monitoring of cell status [9]. Their data indicated that measuring these compounds could be a useful, non-invasive tool for the metabolic monitoring of cells in vitro. Di Somma and co-workers tested the ability of Histogel, a natural mixture of glycosaminoglycans, to sustain the differentiation of ASCs into brown-like cells and brown adipose tissue [12]. A study by Plava and co-workers identified that ASCs are permanently altered in the presence of tumor breast tissue and have the potential to increase tumor cell invasive ability through the activation of epithelial-to-mesenchymal transition in tumor cells [13]. Another study characterized ASCs isolated from patients with rheumatoid arthritis and described their altered phenotype and secretory activity compared to ASCs from healthy donors [14].

In recent years, several in vitro preconditioning (also called pretreatment or licensing) strategies have been investigated to enhance the regenerative and immunomodulatory potential of ASCs. Serejo and co-workers investigated how a preconditioning regime with interferon-γ affects the immunomodulatory functions of ASCs and examined their secretome and released extracellular vesicles [15]. Preconditioned ASCs showed a higher immunosuppressive potential compared to unlicensed ASCs. Another study by our group from the nephrological research laboratory shows in vivo tracking of luciferase-transgenic ASCs after transplantation in a model of inflammatory lung disease [16]. In vivo imaging demonstrated a significantly longer retention time of transplanted ASCs in the injured lung parenchyma compared to healthy wild type mice, which could indicate increased regeneration of the damaged tissue.
